# Supplementary material for: The decrease of intraflagellar transport impairs sensory perception and metabolism in ageing
Source: Nat Commun. 2021 Mar 19;12:1789. doi: 10.1038/s41467-021-22065-8 (PMC7979750; doi:10.1038/s41467-021-22065-8)
Supplement: Supplementary file 1 — Supplementary Information [file 41467_2021_22065_MOESM1_ESM.pdf]

## Supplementary information

# Title: The decrease of intraflagellar transport impairs sensory perception and metabolism in ageing

**Authors:** Yincong Zhang[1,2†], Xiaona Zhang[1,2†], Yumin Dai[1,2], Mengjiao Song[1,2], Yifei Zhou[1,2], Jun Zhou[3,4], Xiumin Yan[1,2], Yidong Shen[1,2*]

**Affiliations:**

[1]State Key Laboratory of Cell Biology, Shanghai Institute of Biochemistry and Cell Biology, Center for Excellence in Molecular Cell Science, Chinese Academy of Sciences, 320 Yueyang Rd. 200031 Shanghai, China

[2]University of Chinese Academy of Sciences, 100049 Beijing, China

[3]Institute of Biomedical Sciences, College of Life Sciences, Key Laboratory of Animal Resistance Biology of Shandong Province, Collaborative Innovation Center of Cell Biology in Universities of Shandong, Shandong Normal University
88 East Wenhua Rd. 250014 Jinan, Shandong, China

[4]State Key Laboratory of Medicinal Chemical Biology, College of Life Sciences, Nankai University, 94 Weijin Road, 300071 Tianjin, China

†These authors contributed equally: Yincong Zhang, Xiaona Zhang

*Correspondence to: yidong.shen@sibcb.ac.cn.

Tel: +86-21-54921171

The **Supplementary Information** file contains eight figures.

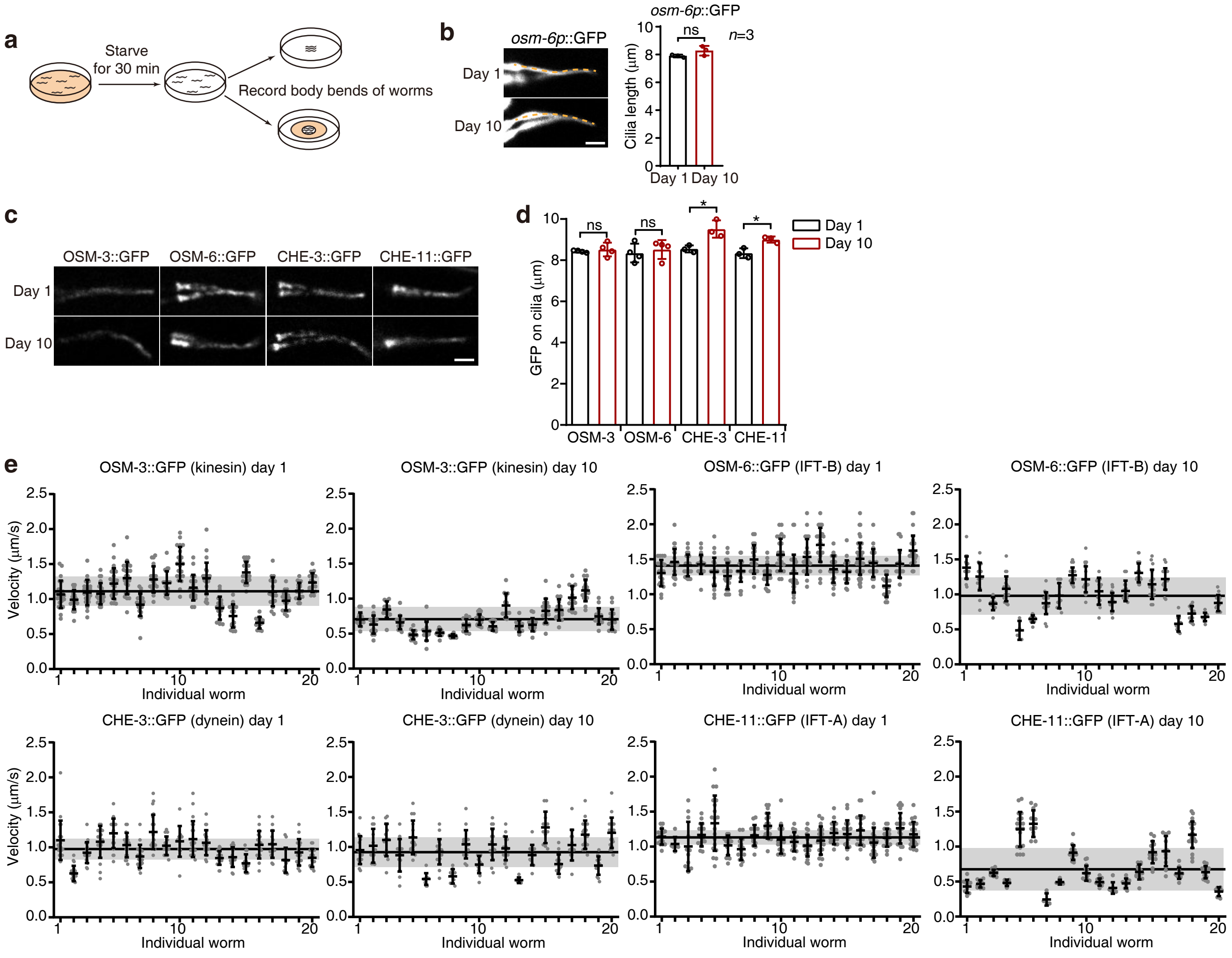


**Supplementary Fig. 1. The intraflagellar transport (IFT) is decreased in aged WT worms.**

**a.** A depiction of the enhanced slowing response assay. After 30 min of starvation, worms were transferred to either an empty plate or to the centre of a ring-like bacteria lawn. Worms body bends were recorded 30 min later. Orange denotes bacteria.

**b.** Cilia length in young and aged worms by a diffuse GFP (osm-6p::GFP) as cilia markers. Dotted lines denote the cilia. Scale bar: 2 µm. *n* =3 biological independent experiments. Exact sample size and *p* value are included in Source Data file.

**c-d.** Cilia length in young and aged worms. Indicated IFT proteins were endogenously tagged with GFP and used as cilia markers. Scale bar: 2 µm. *n* =4 biological independent experiments in OSM-3 and OSM-6, or *n* =3 biological independent experiments in CHE-3 and CHE-11. Exact sample size and *p* value are included in Source Data file.

**e.** The velocities of the indicated IFT components are increasingly varied among individual worms with ageing. For each IFT component, 20 worms randomly picked from three biological replicates are shown. The solid lines and the grey areas denote the mean and the stand deviation of IFT velocities, respectively.

Data are presented as mean ± SD. Unpaired *t*-test (two-tailed), * $p<0.05$, ns: non-significant. Source data are provided as a Source Data file.

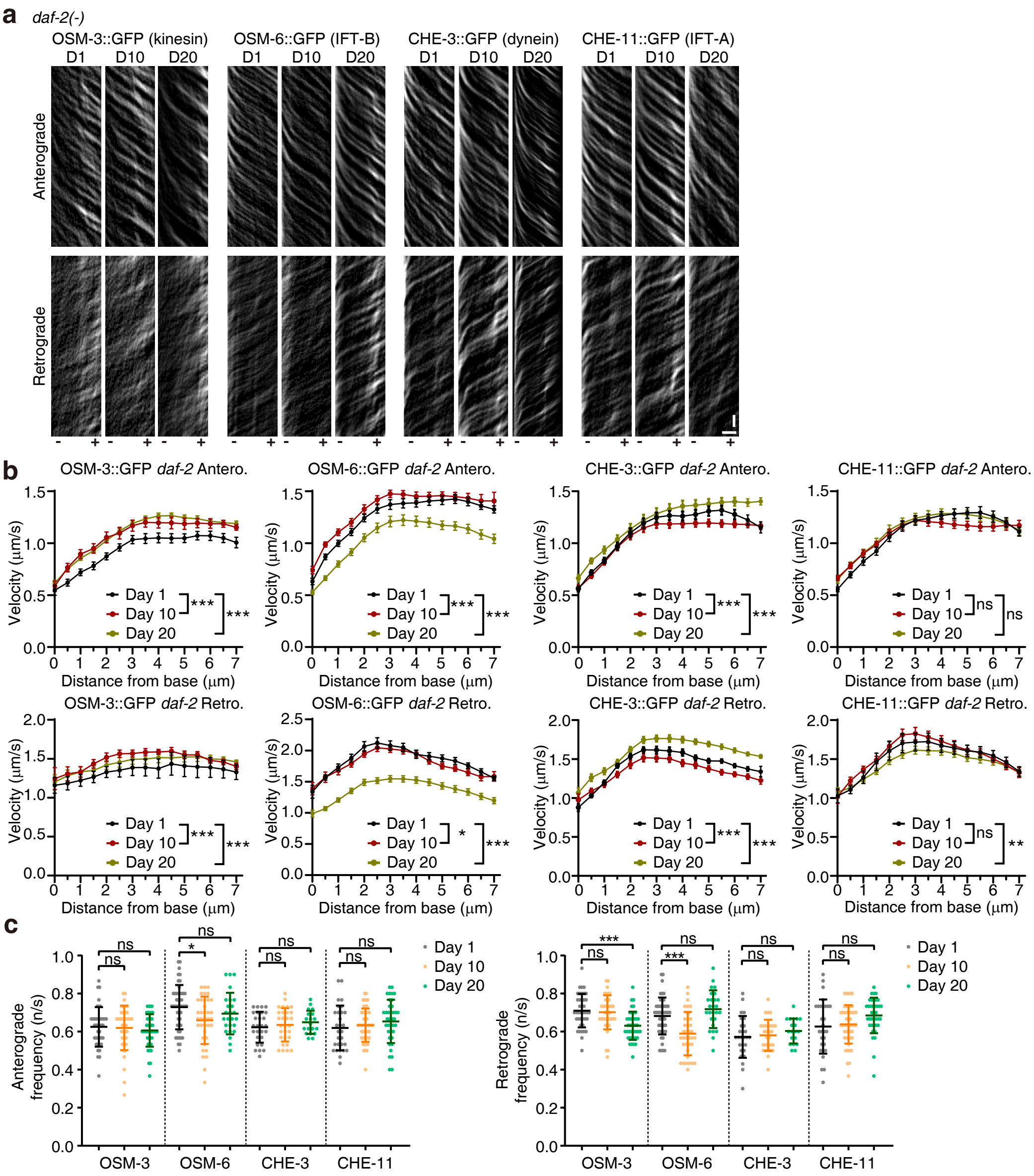


**Supplementary Fig. 2. The longevity mutant of *daf-2(-)* maintains IFT during ageing.**

**a.** The representative kymographs of indicated IFT components in the sensory cilia of *daf-2(-)* mutants at day 1, day 10, and day 20 of adulthood. Similar results were obtained in all independent experiments. These worms were examined in parallel with the WT worms shown in Fig. 1d-f. (+) and (-) denote microtubule polarity. Horizontal scale bars: 2 µm; vertical scale bars: 2 s.

**b-c.** The velocities (b) and frequencies (c) of the indicated IFT components are well maintained during ageing in *daf-2(-)* mutants. Exact sample size and *p* value are included in Source Data file. IFT velocities are show as mean ± SEM, IFT frequencies are show as mean ± SD. Two-way ANOVA in **(b)**, unpaired *t*-test (two-tailed) in **(c)**, * $p$<0.05, ** $p$<0.01, *** $p$<0.0001, ns: non-significant. Source data are provided as a Source Data file.

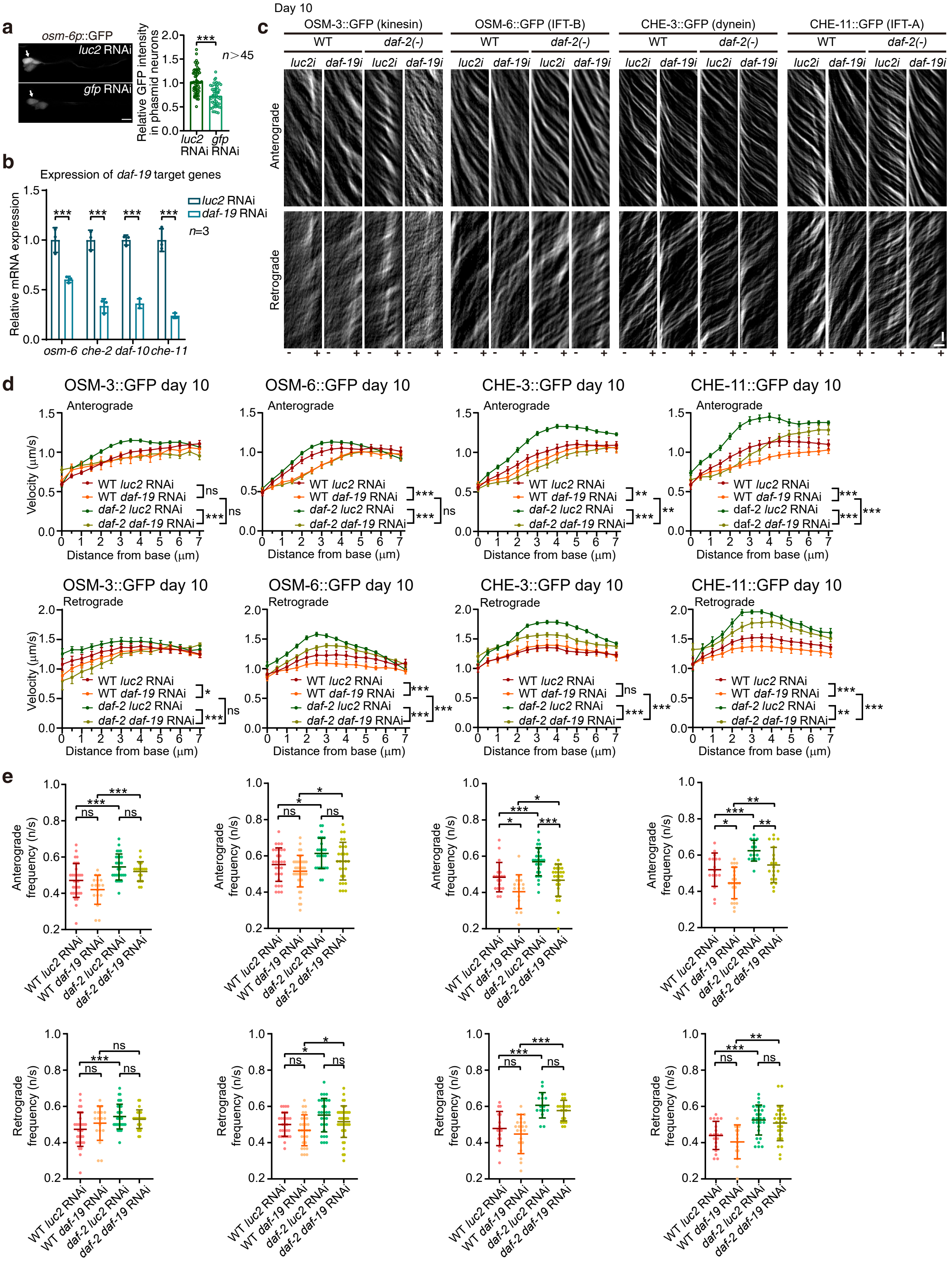

a
osm-6p::GFP
luc2 RNAi
gfp RNAi
Relative GFP intensity in phasmid neurons
***
n>45
luc2 RNAi
gfp RNAi
b
Expression of daf-19 target genes
Relative mRNA expression
luc2 RNAi
daf-19 RNAi
n=3
osm-6
che-2
daf-10
che-11
c
Day 10
OSM-3::GFP (kinesin)
OSM-6::GFP (IFT-B)
CHE-3::GFP (dynein)
CHE-11::GFP (IFT-A)
WT
daf-2(-)
luc2i
daf-19i
Anterograde
Retrograde
d
OSM-3::GFP day 10
OSM-6::GFP day 10
CHE-3::GFP day 10
CHE-11::GFP day 10
Anterograde
Retrograde
Velocity (μm/s)
Distance from base (μm)
WT luc2 RNAi
WT daf-19 RNAi
daf-2 luc2 RNAi
daf-2 daf-19 RNAi
e
Anterograde frequency (n/s)
Retrograde frequency (n/s)
WT luc2 RNAi
WT daf-19 RNAi
daf-2 luc2 RNAi
daf-2 daf-19 RNAi

**Supplementary Fig. 3. The longevity mutant of *daf-2(-)* maintains IFT during ageing through DAF-19/RFX.**

**a.** GFP quantification in phasmid neurons under neuron-specific *gfp* RNAi.

**b.** The relative mRNA expressions of *daf-19* target genes at day 10 under neuron-specific *daf-19* RNAi. *n* =3 biological independent experiments. Exact sample size and *p* value are included in Source Data file.

**c.** The representative kymographs of indicated IFT components in the sensory cilia at day 10 of adulthood. Similar results were obtained in all independent experiments. (+) and (-) denote micro-tubule polarity. Horizontal scale bars: 2 μm; vertical scale bars: 2 s.

**d-e.** The neuron-specific RNAi against *daf-19* abrogates the enhanced IFT in the *daf-2(-)* mutants at day 10 of adulthood. Worms undergoing mild RNAi treatment with normal cilia length were examined. Velocities and frequencies are respectively shown in (d) and (e). Exact sample size and *p* value are included in Source Data file.

IFT velocities are show as mean ± SEM, the rest data are shown as mean ± SD. Two-way ANOVA in **(d)**, Unpaired *t*-test (two-tailed) in the rest, * $p$<0.05, ** $p$<0.01, *** $p$<0.0001, ns: non-significant. Source data are provided as a Source Data file.

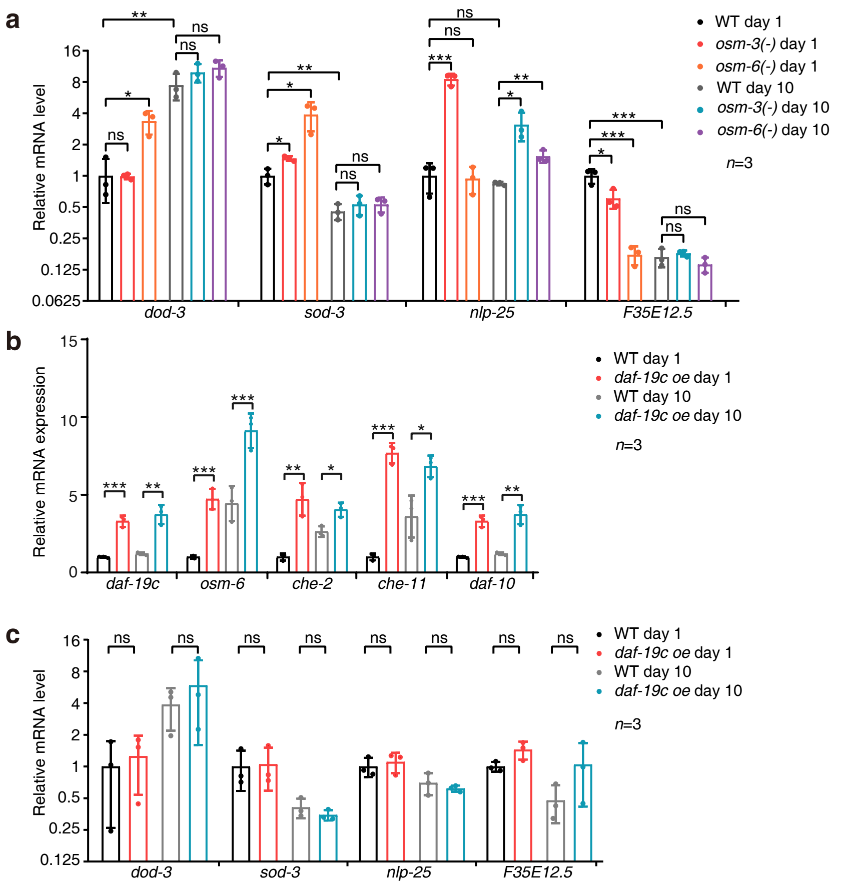


**Supplementary Fig. 4. The insulin/IGF-1 signalling (IIS) is affected by the inhibition but not the enhancement of sensory perception.**

**a.** mRNA levels of the indicated IIS targets in the indicated strains at day 1 and day 10 of adulthood. *n* =3 biological independent experiments.

**b.** Overexpressing *daf-19c* upregulates *daf-19c* and its target genes. *n* =3 biological independent experiments.

**c.** Overexpressing *daf-19c* does not change the expression of the indicated IIS targets at indicated ages. *n* =3 biological independent experiments.

Data are presented as mean ± SD. Unpaired *t*-test (two-tailed), * $p$<0.05, ** $p$<0.01, *** $p$<0.001, ns: non-significant. Source data with exact *p* values are provided as a Source Data file.

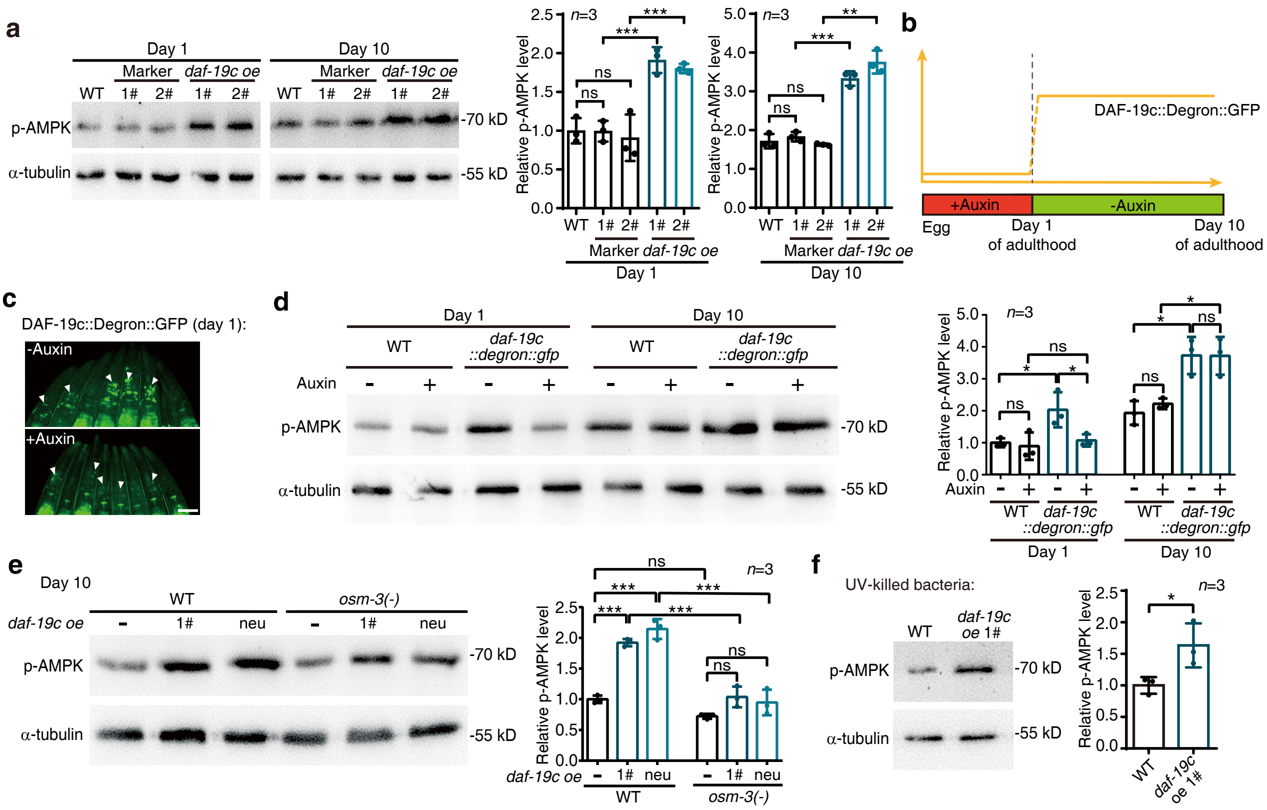


**Supplementary Fig. 5. Overexpressing *daf-19c* upregulates AMPK signalling.**

**a.** Co-injection markers do not change the p-AMPK levels in worms. Marker 1# (*egl-17p::mCherry*) and 2# (*myo-3p::cfp*) denote the worms with the fluorescent markers in *daf-19c oe* 1# and 2#, respectively. *n* =3 biological independent experiments.

**b.** The strategy to induce *daf-19c* overexpression specifically in adulthood.

**c.** DAF-19c::Degron::GFP is effectively inhibited by auxin. Similar results were obtained in all independent experiments. Worms were examined at day 1 of adulthood right before collecting for p-AMPK assays. Arrow heads denote the neurons where DAF-19c::Degron::GFP is expressed. Scale bar: 50 μm.

**d.** The p-AMPK levels of the indicated strains at day 1 and 10 of adulthood. *n* =3 biological independent experiments.

**e.** Disrupting cilia by mutating *osm-3* suppresses the increase of p-AMPK in the worms overexpressing *daf-19c* at day 10 of adulthood by its native promoter (1#) or a neuron-specific promoter (neu). *n* =3 biological independent experiments.

**f.** p-AMPK levels at day 1 of adulthood of the indicated strains fed with UV-killed bacteria. *n* =3 biological independent experiments.

α-tubulin serves as the loading control. Data are presented as mean ± SD. Unpaired *t*-test (two-tailed) in **(f)**, one-way ANOVA in the rest. * $p$<0.05, ** $p$<0.01, *** $p$<0.001, ns: non-significant. Source data with exact $p$ values are provided as a Source Data file.

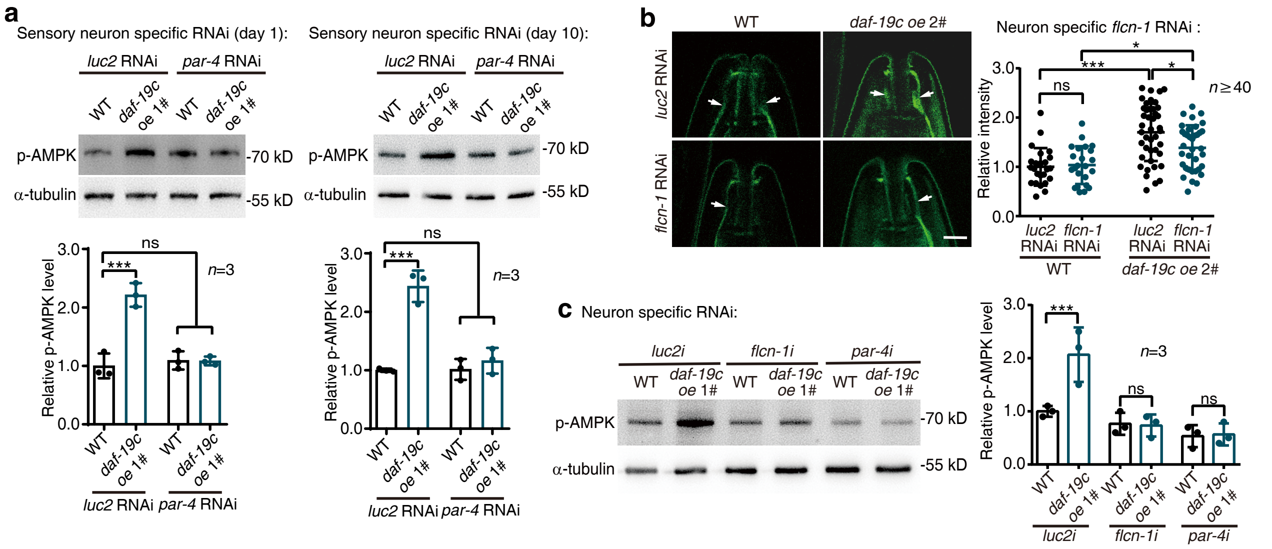


**Supplementary Fig. 6. Overexpressing *daf-19c* upregulates AMPK signalling through PAR-4 and FLCN-1.**

**a.** Sensory neuron specific *par-4* RNAi blocks the upregulation of p-AMPK level in worms overexpressing *daf-19c* both in day 1 and day 10. *n* =3 biological independent experiments.

**b.** The neuron specific RNAi against *flcn-1* blocks the upregulation of PAR-4::GFP on cilia (arrows) in worms overexpressing *daf-19c*. Scale bar: 5 µm.

**c.** The neuron specific RNAi against *flcn-1* blocks the upregulation of p-AMPK in worms overexpressing *daf-19c*. *n* =3 biological independent experiments.

α-tubulin serves as the loading control. Data are presented as mean ± SD. One-way ANOVA. * $p<0.05$, *** $p<0.001$, ns: non-significant. Source data with exact *p* values are provided as a Source Data file.

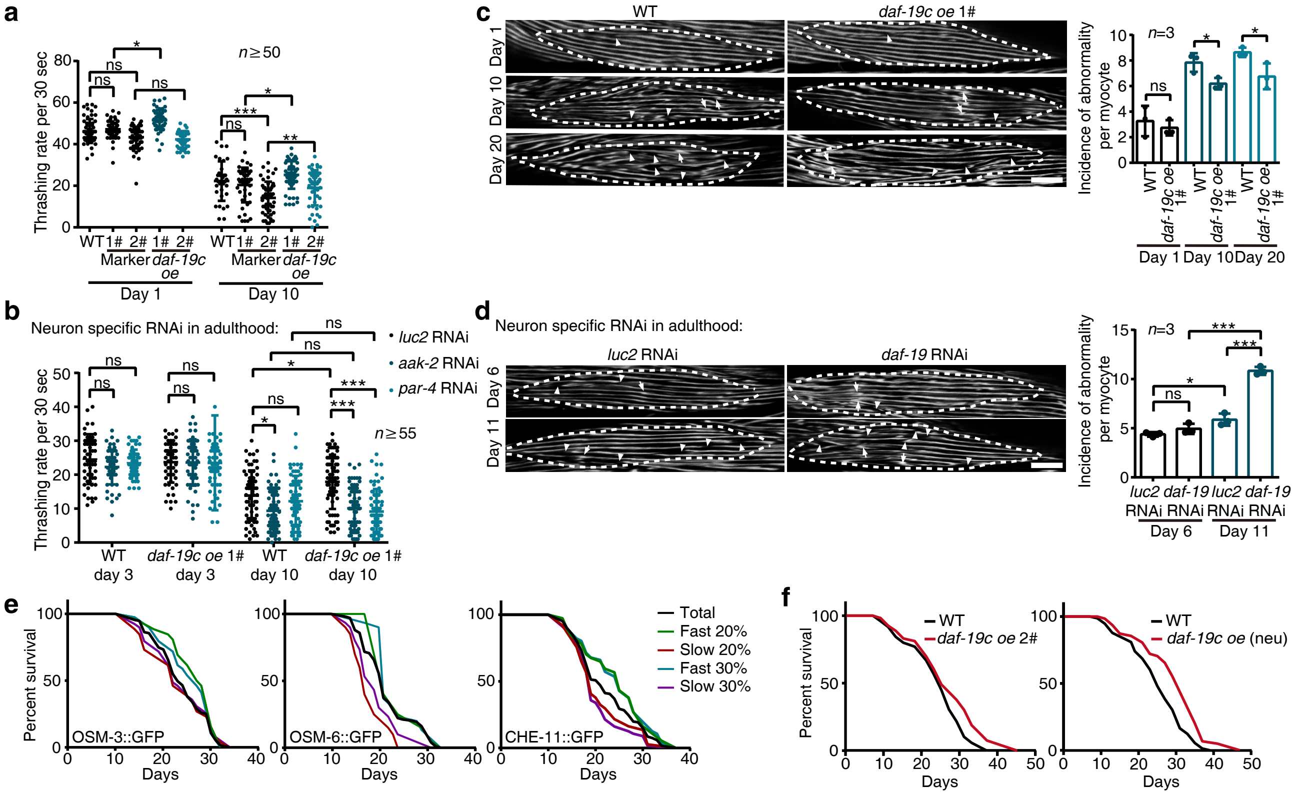


**Supplementary Fig. 7. The enhanced sensory perception promotes the longevity of *C. elegans*.**

**a.** Co-injection markers do not influence the thrashing rates of worms.

**b.** The neuron specific RNAi against *aak-2* or *par-4* in adulthood abrogates the enhanced motility of the aged worms overexpressing *daf-19c*. Note that although the thrashing rates varied from those in Fig. 6b due to the different food source in these two assays, *daf-19c* overexpression consistently increased worm motility.

**c-d.** Overexpressing *daf-19c* (c) suppresses whereas inhibiting *daf-19* in neurons in adulthood (d) promotes the deterioration of myofilaments in aged worms. *n* =3 biological independent experiments. Myofilaments were labelled by MYO-3::GFP. Arrowheads and arrows denote the gaps of myofilaments and the disorganized myofilaments with GFP aggregations, respectively. Scale bars: 25 μm.

**e.** Worms were ranked by the velocities of indicated IFT components from the fastest to the slowest. Fast 20% and 30% are respectively the top 20% and 30% in this rank. The same are to the slow 20% and 30%.

**f.** Overexpressing *daf-19c* with its native promoter (left) or a neuron-specific promoter (right) extends the lifespan of worms.

Data are presented as mean ± SD. One-way ANOVA. * $p$<0.05, ** $p$<0.01, *** $p$<0.001, ns: non-significant. Source data with exact $p$ values are provided as a Source Data file.

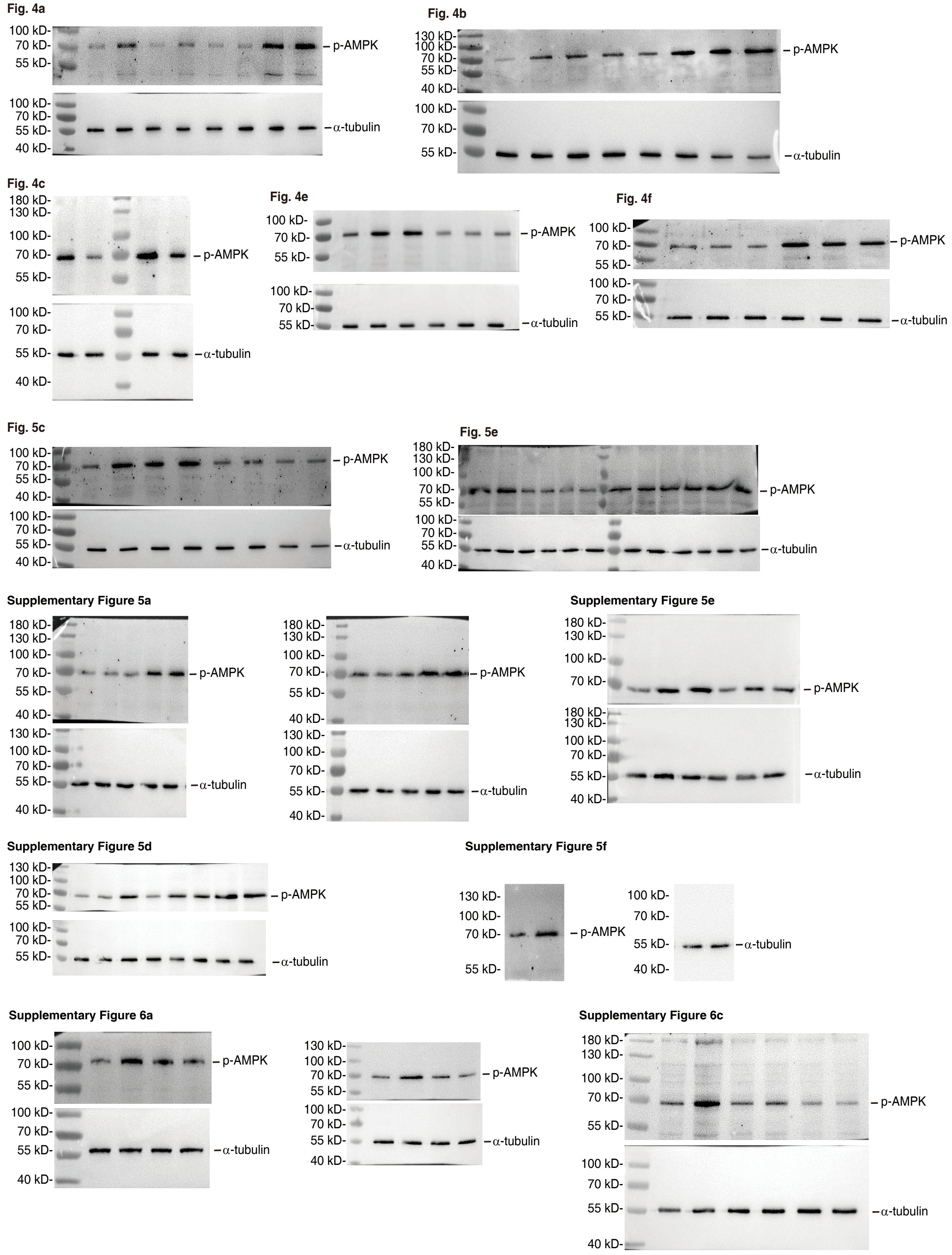


**Supplementary Fig. 8. The uncropped blots of all the presented western blot data.**
