# Supplementary material for: The decrease of intraflagellar transport impairs sensory perception and metabolism in ageing
Source: Nat Commun. 2021 Mar 19;12:1789. doi: 10.1038/s41467-021-22065-8 (PMC7979750; doi:10.1038/s41467-021-22065-8)
Supplement: Supplementary file 3 — Description of Additional Supplementary Files [file 41467_2021_22065_MOESM3_ESM.docx]

**Supplementary Tables**

**Supplementary Table 1.** Worm and bacteria strains.

**Supplementary Table 2.** Primer sequences used in plasmid constructions

**Supplementary Movies**

**Supplementary Movie 1.** The movement of CHE-11::GFP in the phasmid cilia of WT worms at indicated ages. Scale bar: 2 μm.

**Supplementary Movie 2.** The movement of CHE-11::GFP in the phasmid cilia of *daf-2(-)* mutants at indicated ages. Scale bar: 2 μm.

**Supplementary Movie 3.** The movement of CHE-11::GFP in the phasmid cilia of worms overexpressing *daf-19c* at indicated ages. Scale bar: 2 μm.

**Supplementary Movie 4.** The chemotaxis assay of WT and *daf-19c oe* worms at day 5 of adulthood. “B” for the odourant spot with butanone, “N” for nonanone, and “C” for the control spot with ethanol.
