# Supplementary material for: The decrease of intraflagellar transport impairs sensory perception and metabolism in ageing
Source: Nat Commun. 2021 Mar 19;12:1789. doi: 10.1038/s41467-021-22065-8 (PMC7979750; doi:10.1038/s41467-021-22065-8)
Supplement: Supplementary file 10 — Reporting Summary [file 41467_2021_22065_MOESM10_ESM.pdf]

nature research

Corresponding author(s): Yidong Shen
Last updated by author(s): Feb 8, 2021

Reporting Summary

Nature Research wishes to improve the reproducibility of the work that we publish. This form provides structure for consistency and transparency in reporting. For further information on Nature Research policies, see our Editorial Policies and the Editorial Policy Checklist.

Statistics

For all statistical analyses, confirm that the following items are present in the figure legend, table legend, main text, or Methods section.

n/a	Confirmed	
☐	☒	The exact sample size (n) for each experimental group/condition, given as a discrete number and unit of measurement
☐	☒	A statement on whether measurements were taken from distinct samples or whether the same sample was measured repeatedly
☐	☒	The statistical test(s) used AND whether they are one- or two-sided<br>*Only common tests should be described solely by name; describe more complex techniques in the Methods section.*
☐	☒	A description of all covariates tested
☐	☒	A description of any assumptions or corrections, such as tests of normality and adjustment for multiple comparisons
☐	☒	A full description of the statistical parameters including central tendency (e.g. means) or other basic estimates (e.g. regression coefficient) AND variation (e.g. standard deviation) or associated estimates of uncertainty (e.g. confidence intervals)
☐	☒	For null hypothesis testing, the test statistic (e.g. F, t, r) with confidence intervals, effect sizes, degrees of freedom and P value noted<br>*Give P values as exact values whenever suitable.*
☒	☐	For Bayesian analysis, information on the choice of priors and Markov chain Monte Carlo settings
☐	☒	For hierarchical and complex designs, identification of the appropriate level for tests and full reporting of outcomes
☒	☐	Estimates of effect sizes (e.g. Cohen's d, Pearson's r), indicating how they were calculated

Our web collection on statistics for biologists contains articles on many of the points above.

Software and code

Policy information about availability of computer code

Data collection	Time-lapse imaging were carried out on the Ultraview spinning disc confocal (PerkinElmer Ultra VIEW VoX) and fluorescence images were obtained using a confocal microscope(Leica TCS SP8 WLL). Western blots were taken with Tanon chemiluminescence gel imaging system (Tanon-5200Multi).
Data analysis	Images were analyzed via Fiji (version: ImageJ 1.52p) and blots via Adobe Photoshop (version: 13.0). IFT velocities were analyzed by KymographDirect software (version: KymographDirect 2.1). GraphPad Prism software (version: GraphPad Prism 8.0.1) was used to create the graphs.

For manuscripts utilizing custom algorithms or software that are central to the research but not yet described in published literature, software must be made available to editors and reviewers. We strongly encourage code deposition in a community repository (e.g. GitHub). See the Nature Research guidelines for submitting code & software for further information.

Data

Policy information about availability of data

All manuscripts must include a data availability statement. This statement should provide the following information, where applicable:

- Accession codes, unique identifiers, or web links for publicly available datasets
- A list of figures that have associated raw data
- A description of any restrictions on data availability

All data are available within the Article and Supplementary Files, or available from the corresponding authors on reasonable request. Source data are provided with this paper.

Field-specific reporting

Please select the one below that is the best fit for your research. If you are not sure, read the appropriate sections before making your selection.

- [x] Life sciences
- [ ] Behavioural & social sciences
- [ ] Ecological, evolutionary & environmental sciences

For a reference copy of the document with all sections, see nature.com/documents/nr-reporting-summary-flat.pdf

Life sciences study design

All studies must disclose on these points even when the disclosure is negative.

Sample size	Sample size and statistical test were chosen based on previous studies that used the same or similar methods.<br>For IFT motility: Prevo et al. Nature Cell Biology 17, 1536-1545 (2015)<br>For fluorescence microscopy: Monje et al. Aging Cell 10, 1921-1031 (2011)<br>For Western blot: Yifei et al. Nature communications 10, 1-14 (2019)<br>For trashing assays: van Ham TJ, et al. Cell 142, 601"612 (2010).<br>For longevity: Hsin H and Kenyon. C. Nature 399, 362"366 (1999).<br>For chemiotaxis: Bargmann et al. Neuron 7, 129-142 (1991)<br>Please see the figure legends and supplementary information for the precise number of replicates.
Data exclusions	For lifespan assays, the worms undergoing internal hatching, bursting vulva, crawling off the plates, or contamination were censored. For time-course microscopy, the data of some time points were excluded due to contamination. The exclusion criteria were pre-established.
Replication	All data in the manuscript was obtained with at least three biological replicates.
Randomization	All samples/worms were allocated into experimental groups randomly.
Blinding	Blind group allocation was done for: Intensity measurements, fluorescent images and ageing assays as they may be subjected to human error. All other experiments were not blinded as they rely on objective instrument measurements such as for the qPCR analysis, IFT motility analysis.

Reporting for specific materials, systems and methods

We require information from authors about some types of materials, experimental systems and methods used in many studies. Here, indicate whether each material, system or method listed is relevant to your study. If you are not sure if a list item applies to your research, read the appropriate section before selecting a response.

Materials & experimental systems

n/a	Involved in the study	
[ ]	[x]	Antibodies
[x]	[ ]	Eukaryotic cell lines
[x]	[ ]	Palaeontology and archaeology
[ ]	[x]	Animals and other organisms
[x]	[ ]	Human research participants
[x]	[ ]	Clinical data
[x]	[ ]	Dual use research of concern

Methods

n/a	Involved in the study	
[x]	[ ]	ChIP-seq
[x]	[ ]	Flow cytometry
[x]	[ ]	MRI-based neuroimaging

Antibodies

Antibodies used	Monoclonal Anti-α-Tubulin Antibody Produced in Mouse (Sigma-Aldich, Cat# T5168);<br>Monoclonal Anti-p-AMPK Antibody Produced in Rabbit (CST, Cat# 4188s).<br>Goat Anti-Rabbit IgG (H+L) Secondary Antibody, HRP (Thermo Fisher Scientific, Cat# G-21234)<br>Goat Anti-Mouse IgG (H+L) Secondary Antibody, HRP (Thermo Fisher Scientific, Cat# G-21040)
Validation	All antibodies were used for western blotting in current study.<br>Anti-α-Tubulin(Mouse Monoclonal), manufacture's description:https://www.sigmaaldrich.com/catalog/product/sigma/t5168?lang=zh&region=CN<br>Anti-p-AMPK(Rabbit Monoclonal),manufacture's description:https://www.cellsignal.com/products/primary-antibodies/phospho-ampka-thr172-d79-5e-rabbit-mab/4188

Animals and other organisms

Policy information about studies involving animals; ARRIVE guidelines recommended for reporting animal research

Laboratory animals	C. elegans hermaphrodites throughout their lifespan were used in this study. The detailed information of all worm strains were listed in the Supplementary Table 1.
Wild animals	No wild animals were used in this study.
Field-collected samples	No field-collected samples were used in this study.
Ethics oversight	No ethical approval or guidance was required in this study.

Note that full information on the approval of the study protocol must also be provided in the manuscript.
